# Segmenting sign language into motor primitives with Bayesian binning

**DOI:** 10.3389/fncom.2013.00068

**Published:** 2013-05-27

**Authors:** Dominik Endres, Yaron Meirovitch, Tamar Flash, Martin A. Giese

**Affiliations:** ^1^Department of Cognitive Neurology, Section Computational Sensomotorics, CIN, HIH and University Clinic TübingenTübingen, Germany; ^2^Department of Applied Mathematics and Computer Science, The Weizmann Institute of ScienceRehovot, Israel

**Keywords:** motor primitives, two-thirds power law, differential invariants, Bayesian binning, sign language, minimum jerk model

## Abstract

The endpoint trajectories of human movements fulfill characteristic power laws linking velocity and curvature. The parameters of these power laws typically vary between different segments of longer action sequences. These parameters might thus be exploited for the unsupervised segmentation of actions into movement primitives. For the example of sign language we investigate whether such segments can be identified by Bayesian binning (BB), using a Gaussian observation model whose mean has a polynomial time dependence. We show that this method yields good segmentation and correctly models ground truth kinematics composed of consecutive segments derived from wrist trajectories recorded from users of Israeli Sign Language (ISL). Importantly, polynomial orders between 3 and 5 yield an optimal trade-off between complexity and accuracy of the trajectory approximation, in accordance with the minimum acceleration and minimum jerk models. Comparing the orders of the polynomials best approximating natural kinematics against those needed to fit the power law ground truth data suggests that kinematic properties not compatible with power laws are also not adequately represented by low order polynomials and require higher order polynomials for a good approximation.

## 1. Introduction

Complex motor behavior might be organized in terms of sequences of temporal movement primitives that follow each other sequentially in time. Determining such primitives from kinematic data is an important problem for many technical applications, e.g., in robotics, computer vision and computer graphics. At the same time, the characterization of possible temporal primitives that underlie the planning and execution of complex motor behavior remains a partially unresolved issue in motor control (Flash and Hochner, 2005). While the appropriate characterization of the temporal organization of complex motor behavior might require ultimately hierarchical multi-level representations (Flash and Hochner, 2005), many previous studies that investigated the nature of such primitives have focused on the analysis of movement kinematics. Specifically, it has been investigated how the temporal and kinematic properties of the movement are influenced by the path followed by the hand (see e.g., Polyakov et al., 2009b).

One approach to the definition of temporal segments is based on invariant properties that characterize movements within individual segments. It was already established at the end of the nineteenth century that for arm movements, curvature and speed are correlated variables, speed typically obeying an inverse relation to curvature (Jack, 1895). Almost a 100 years later, this rule was quantitatively formalized as the two-thirds power law. Specifically, this rule dictates that for figure drawing movements the speed along the motion path is proportional to the curvature of this path raised to the minus one-third power (Lacquaniti et al., 1983):

(1) $$\left|v(t)\right|=\alpha \kappa^{-\frac{1}{3}}(t)$$

where *v* is the Euclidean velocity, κ is the Euclidean curvature (i.e. the reciprocal of the radius of osculating circle) and α is the so called velocity gain factor, which is constant within each individual segment.

Extensive research has investigated the conditions and origins of the two-thirds power law. Equation (1) was first developed for two-dimensional drawings but was also later applied to three-dimensional drawing under isometric force conditions (Massey et al., 1992) and to various movement modalities including eye pursuit (Viviani and deSperati, 1997) and speech movements (Tasko and Westbury, 2004; Perrier and Fuchs, 2008). Also, the exponent of the two-thirds power law varies in children and becomes more stable with age (Viviani and Schneider, 1991). This principle not only applies to motion production but also to motion perception as has been supported by studies of the perception of handwriting and drawing movements (Soechting et al., 1986; Viviani et al., 2000) and of the motion of abstract visual stimuli (Viviani and Stucchi, 1992; Levit-Binnun et al., 2006). Finally, a functional magnetic resonance imaging (fMRI) study has supported a central representation of the perception of this kinematic law (Dayan et al., 2007). Taken together, these studies show that the 2/3 power law is most likely not the expression of bio-mechanical constraints but may reflect the involvement of the central nervous system (for a review see Flash et al., 2012).

A recent study (Meirovitch, 2008) has investigated the wrist trajectories in sign language with the goal of identifying the motor control strategies for such spatially constrained movements. Movement recordings from normal naive participants revealed that a generalized form of the 2/3 power law (Equation 1) predicts the velocity profiles of the wrist trajectories. The executed trajectories across a number of repetitions were fitted with the model:

(2) $$\left|v(t)\right|=\alpha \kappa^{\beta }(t)$$

where *v*, κ and α are defined in the same way as for Equation (1) and the parameter β ∈ [−1, 0] typically remains constant during individual trajectory segments. This suggests that this parameter might be used to segment longer action sequences into movement primitives, identifying the segments with an approximately constant β. The presence of the kinematic segments does not necessarily imply segmented control by the brain [see Schaal and Sternad (1999) and Flash and Hochner (2005)]. Therefore, an attempt to unravel kinematic primitives would require to be consistent with optimization models used in motor control such as the minimization of jerk (time derivative of acceleration), variance, etc. [see section 1.1]. Here, we employ the segment-wise constancy of β to generate ground-truth data for the testing of motion segmentation algorithms. We use sign language trajectories as a basis for our analysis and modify their velocity to match a possible power law segmentation with fixed values of the parameters α and β within each predefined segment. The timing of each segment in the ground-truth parameterization closely follows the timing of the natural sign language trajectories. Moreover, in simulating the discrete segmentation of the ground-truth data, the algorithm optimizes for a smooth transition between adjacent segments by choosing suitable power law parameters. The details of this procedure are described in section 2.4.

### 1.1. Connection between polynomials and power laws

In addition to these power laws (Equation 2), human movements were shown to be well-captured by optimization models that maximize the smoothness of the trajectories, mathematically expressed by the minimization of integrated jerk or by higher-order time derivatives of position, i.e., snap, crackle and so on (Flash and Hogan, 1985; Todorov and Jordan, 1998). Other types of movements (e.g. locomotion and arm reaching) were shown to be well captured by minimum acceleration models (Ben-Itzhak and Karniel, 2008; Mombaur et al., 2010). Mathematically, such models predict that the trajectories will be well captured by polynomials of orders 3, 5, and 7, corresponding to minimum acceleration, jerk and snap models, respectively. In Richardson and Flash (2002) it was mathematically shown that such polynomial trajectories, which optimize mean squared derivative cost functions

(3) $$C_{n}=\int_{0}^{T}{\left\|\frac{d^{n}r}{dt^{n}}\right\|}^{2}dt,$$

(where *n* = 3, 4 correspond to minimum jerk and snap, respectively) follow generalized power laws whose exponents depend on the cost function being optimized and on the geometrical shape of the trajectory being traced. In addition, such predicted power laws were shown to be consistent with the power law found empirically in the experimental data.

In another study Polyakov et al. identified parabolic strokes whose generation both obeys the 2/3 power law and yields minimum-jerk trajectories (Polyakov et al., 2009b). Parabolas are interesting because of their invariance with respect to affine transformations and additionally their special role as geodesics in equi-affine geometry which predicts the two-thirds power law (Flash and Handzel, 2007).

For 3-dimensional geometrically complex trajectories, a power law that depends on torsion which measures the rate of change of the osculating plane, was analyzed for 3D drawing movements (Maoz et al., 2009; Pollick et al., 2009) and although the link between power laws and variational optimization principles was studied for several figural forms (Polyakov et al., 2009b), such links have not been examined for natural complex trajectories. Here we provide the first detailed account for the computational equivalence between the generalized power law and variational models. To this end, we present a Bayesian approach for the temporal segmentation of complex end-effector trajectories based on a polynomial observation model and show that the resulting segments can be used to identify the power law structure of the kinematic profiles.

### 1.2. Unsupervised segmentation of complex end-effector movements

Segmenting trajectories into power-law obeying pieces is difficult for three reasons: first, the number of segments and their temporal boundaries are *a-priori* unknown. Second, estimating higher derivatives from noisy trajectory data is prone to errors. Third, segments obeying power laws with different βs (Equation 2) are typically connected by short transition periods during which the trajectory is not well described by a power law (Viviani and Flash, 1995). We address the first two problems by choosing a Bayesian approach, based on Bayesian Binning (BB). Estimating the number of segments is a model complexity estimation problem, which we deal with using the “Occam's razor” inherent in Bayesian approaches: the larger parameter space of more complex models (e.g., having more segments) implies that every individual instantiation of such a model is *a-priori* less probable than a more parsimonious model (Bishop, 2007). Thus, simpler models are preferable, if they can explain the observable data equally well. To handle the (possibly large amounts of) noise in parameter estimates, Bayesian models infer a posterior distribution over parameters, instead of point estimates yielded by maximum-likelihood fitting procedures. This posterior distribution allows to assess not only the expected value, but also the uncertainty of the parameter of interest. We sidestep the third problem—transition periods which do not obey power laws—by using a dataset in which all transition periods are very short, almost instantaneous, and a segment model which can also well describe the point to point and transition movements (Flash and Hogan, 1985; Polyakov et al., 2009a). As detailed above, both polynomials and power laws can be derived from the same optimization principles, and power law trajectories can be well fitted by time-dependent polynomials, but the kinematic profiles at the boundaries of segments are better explained by polynomials. Furthermore, the parameterization by polynomials avoids singularities that arise in Equation (2) for straight curve segments with zero curvature.

The rest of the paper is structured as follows. First we give a concise description of the data recordings and ground truth generation in section 2, since these data have not been published before. We develop BB for the segmentation of wrist trajectories recorded with motion capture in section 2.5. There, we show how to use BB with observations models having a polynomial time dependence of the mean and segment-wise constant coefficients. In section 3 we demonstrate the results achieved by BB and compare them with the ground truth. Finally, we give an outlook for further investigations in section 4.

## 2. Materials and methods

To generate a form of ground truth data that complies with the kinematic law (Equation 2), we corrected the speed parameterization of the original, recorded trajectories in a way that made them exactly compatible with the kinematic law.

Our experiments were based on data adapted from Meirovitch (2008). A short description of the experiments is given below. In section 2.4 we give a more detailed description of the segmentation mechanism which we used to synthesize the “ground truth” segmentation. While the employed synthesis method may appear complicated, we chose it to generate a ground truth with a high degree of biological realism.

### 2.1. Subjects and signs

Subjects were two natural users of Israeli Sign Language (ISL): S00 (male, 45), a native signer who acquired ISL during his childhood through exposure to his parents, and S01 (male, 44) who acquired ISL in childhood.

Each subject was asked to sign two words, either “cake (baked)” or “chandelier.” The English equivalents of these ISL signs were shown in English on a screen in front of the subject prior to execution. Each sign was repeated 20 times.

### 2.2. Data recording and preprocessing

Hand movements were recorded using the Polhemus LIBERTY 240/16 motion capture system which recorded the location of a sensor fixed to the subject's wrist at an accuracy of 0.08 cm at a frequency of 240 Hz.

The trajectories were preprocessed with a 50-samples 6 Hz low pass FIR filter (normalized gain of −6 dB at 6 Hz), and their velocity profiles and Euclidean curvature were calculated for each sample *n* (Calabi et al., 1998).

### 2.3. Fitting of power law and quantification of compliance

We used correlation coefficients to compare the actual trajectories with the predictions based on the best-fitting power law within individual trajectory segments. Within each segment, the law was fitted using non-linear regression (Coleman and Li, 1994, 1996; Maoz, 2007), where the predicted speed value is denoted as $\hat{v}(n)$ and where $\hat{\alpha }$ and $\hat{\beta }$ are the fitted parameter values for this segment. The predicted speed is given by the relationship:

(4) $$\hat{v}(n)=\hat{\alpha }\kappa^{\hat{\beta }}(n)$$

A measure for the quality of the fit is given by the compliance

(5) $$R_{s}^{2}(\hat{\alpha },\;\hat{\beta })=1-\frac{\sum_{i\leq n\leq j}{\left(v(n)-\hat{v}(n)\right)}^{2}}{\sum_{i\leq n\leq j}{\left(v(n)-v_{\text{average}}\right)}^{2}}$$

where *s* is the segment for which regression is carried out and *v*_average_ is the average speed within the segment.

### 2.4. Power law segmentation

To generate a data set as ground truth that fully complies with the power law (Equation 2) the speed of the original trajectories was reparameterized (by time warping) to make the individual trajectory segments exactly compatible with the best fitting kinematic law. The purpose of the time-warping is the generation of a ground truth dataset that fully complies with the power law against which the automatic (polynomial) segmentation can be compared. Our paradigm enables the treatment of several minimization principles (e.g., acceleration, jerk, snap, crackle etc.) in parallel by choosing the model via Bayesian model comparison.

**Figure 1:**
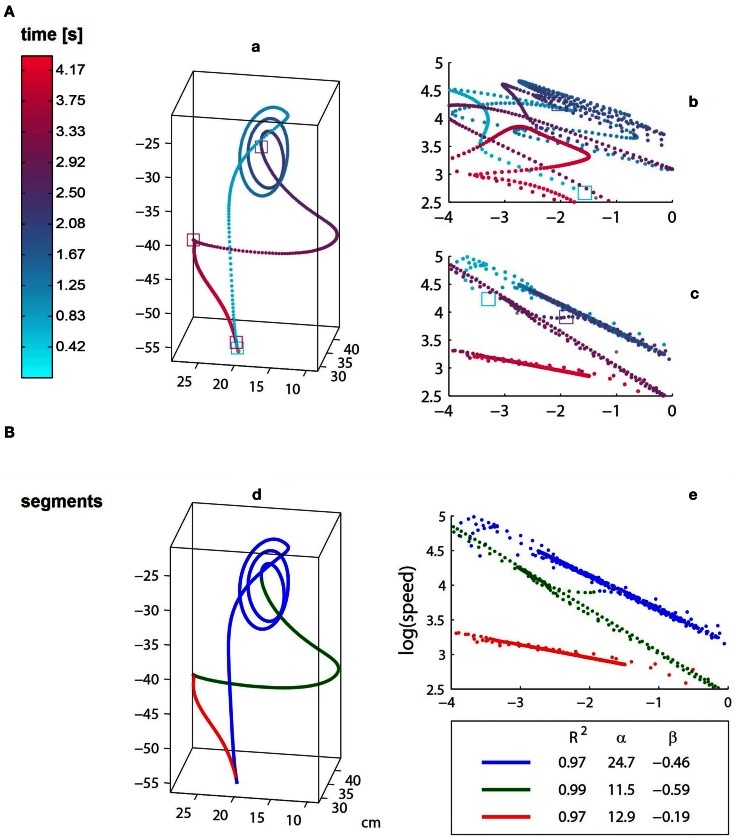
**The trajectories of an ISL “Cake” repetition before **(a)** and after **(d)** time-warping. (A)** The samples of the raw data are colored according to time. **(B)** The time-warped trajectory, where the three colors designate power law segments. Log–Log plots of curvature and velocity are depicted on the right side: **(b)** raw data and **(c,e)** time-warping, colored according to time and segments, respectively. It can be seen that the spatial representation, i.e., path, of the raw and time-warped trajectories are identical. Also, although both raw and time-warped log–log curves are characterized by linear segments, the time warping is based on a simpler power law representation which is characterized by three highly fitting (*R*^2^ > 0.97) segments with beta values ranging from about −0.19 to −0.6. The transitions between long straight segments in the log–log representation are made either by a very brief transitional period which does not comply with the power laws of the adjacent segments [e.g., the portion between the blue and green segments in **(e)**] or in the temporal point of intersection of the piecewise linear sections [e.g., green to red segment in **(e)**].

The idea employed in the ground truth synthesis is based on randomizing some parameters of the segmentation while optimizing others. At the first step the algorithm iterates through possible segmentations in which the temporal breakpoints and the exponents of the power laws are randomized. To respect the smoothness characteristic of natural movement, the gain-factors of the respective segments are then calculated from the curvatures at the boundaries of the segments, and the trajectory is then time-warped. The segments that comprise the ground truth dataset have durations comparable to the respective segments in the natural trajectories, comparable maximal speed, and their speed is continuous at the boundaries. An example of a synthesized ground truth trajectory is shown in Figure 1.

In the following, we give some additional technical details: To avoid singularities we excluded from the data basis the initial and terminal parts of the movement, where the speed was below a prescribed ratio of the maximal speed (<15% × maximal speed).

First, each trajectory was randomly partitioned into *N* = 3 consecutive time intervals, {[*t*_*i*_, *t*_*i* + 1_]}^*N*^_*i* = 1_, where the first interval begins at *t*_1_, and the last interval terminates at the end of the recorded movement, at time *t*_*N* + 1_, such that the duration of each segment, *t*_*i* + 1_ − *t*_*i*_, was not too short (>300 ms). We proceeded to synthesize the power law parameters for each of the random partitions. At the first step, we uniformly randomized β = {β_1_, …, β_*N*_} ∈ [−1, 0]^*N*^ such that |β_*i* + 1_ − β_*i*_| > 0.1 and those β *N*-tuples that were biologically implausible were rejected according to criteria that are described below.

The α parameter, α = {α_1_, …, α_*N*_}, was determined based on the randomized β value and the empirical speed and curvature. The first value, α_1_, was determined from the empirical speed using Equation (2) according to:

$$\alpha_{1}=v(0)\kappa {\left(0\right)}^{-\beta_{1}},$$

The α_*i* + 1_ value was determined enforcing the constraint that the speed should be continuous at the segment boundaries, resulting in the relationship:

$$v(t_{i+1})=\alpha_{i+1}\kappa {\left(t_{i+1}\right)}^{\beta_{i+1}}=\alpha_{i}\kappa {\left(t_{i+1}\right)}^{\beta_{i}}.$$

Using spline interpolation we reparameterized the trajectories within each time interval, [*t*_*i*_, *t*_*i* + 1_], defining a new effective time parameter, τ, that was defined up to a constant by:

$$ds=\alpha_{i}\kappa {\left(t\right)}^{\beta_{i}}d\tau$$

where *ds* is the Euclidean arc-length parameterization of the trajectory. This relationship results in the differential equation:

$$\frac{d\tau }{dt}=\frac{1}{\alpha_{i}}v(t)\kappa {\left(t\right)}^{-\beta_{i}}$$

that links the original and the warped time axis of the trajectory.

Finally, those trajectories that induced biologically improbable high speed ratios, were rejected. For those synthesized trajectories that were not rejected, we recalculated, using non-linear regression (see section 2.3), the actual $\hat{\alpha }$ and $\hat{\beta }$ *N*-tuples for the accepted [*t*_*i*_, *t*_*i* + 1_] time intervals and those were stored for further analyses. It is important to note that our reparameterization method did not change the durations of the original behavioral time intervals given by τ_*i* + 1_ − τ_*i*_ = *t*_*i* + 1_ − *t*_*i*_.

### 2.5. Bayesian binning for sign language segmentation

In the following, we present an unsupervised segmentation algorithm that is based on BB. Briefly, BB is an approach to modeling data with a totally ordered structure, such as time series, by functions which are piecewise defined. The total order allows for an efficient iteration over all possible segment configurations in polynomial time.

BB was originally developed for modeling of (typically very noisy) neural spike train data (Endres et al., 2008; Endres and Oram, 2009) and their information-theoretic evaluation (Endres and Földiák, 2005). It was later generalized for regression of piecewise constant functions (Hutter, 2007). Concurrently, a closely related Bayesian formalism for dealing with multiple change point problems was introduced by Fearnhead (2006).

To apply BB to wrist trajectories we augment it by an observation model for the trajectory segments which is Gaussian with a full covariance matrix and a polynomial time dependence of the mean. This model was originally developed by two of the authors for segmenting joint angle trajectories of human actors in a “natural” fashion (i.e., in agreement with human intuition) (Endres et al., 2011a,b). To make this paper self-contained, the following sections describe the prior over bin boundaries (section 2.5.1) and the observation model (section 2.5.5). The algorithmic details of evaluating posterior expectations are only outlined schematically, they are elaborated in Endres and Földiák (2005). A full derivation of the polynomial observation model, including the exact posterior updates can be found in Endres et al. (2011a).

The results of this segmentation algorithm are validated using the ground-truth data basis from section 2.4 that consists of trajectories whose segments exactly comply with the previously described power law. We show (section 3) that BB results in good segmentation of data fulfilling this kinematic law. Furthermore, we argue that BB generalizes the segmentation approaches presented in Barbič et al. (2004) and Polyakov et al. (2009b) [see section 4].

#### 2.5.1. The bin boundary prior

**Figure 2:**
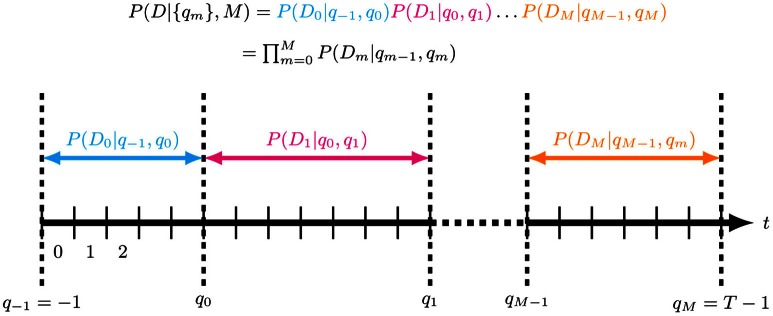
**Segmentation of a time series of length *T* into *M*+ *1* contiguous, non-overlapping segments with (inclusive) upper segment boundaries *q*_*m*_ ∈ {*q*_*m*_}.** The observation model in each segment *m* is given by *P*(*D*_*m*_|*q*_*m* − 1_, *q*_*m*_), where *D*_*m*_ is that part of the data which is in segment *m*. Importantly, the model assumes that the data are independent across segments given the {*q*_*m*_} and *M*.

Our objective is to model a time series *D* in the time interval [*t*_min_ = *t*_1_, *t*_max_ = *t*_*N* + 1_]. We discretize [*t*_min_, *t*_max_] into *T* contiguous intervals of duration Δ*t* = (*t*_max_ − *t*_min_)/*T*, such that interval *j* is [*j* · Δ*t*, (*j* + 1) · Δ*t*] (see Figure 2). Choose Δ*t* small enough to capture all relevant features of the data^1^. We model the generative process of *D* by *M* + 1 contiguous, non-overlapping segments, indexed by *m* and having *inclusive* upper boundaries *q*_*m*_ ∈ {*q*_*m*_}. The segment *m* therefore contains the time interval *T*_*m*_ = (Δ*t q*_*m* − 1_, Δ*t q*_*m*_]. Let *D*_*m*_ be that part of the data which falls into segment *m*. We assume that the probability of *D* given {*q*_*m*_} factorizes as

(6) $$P(D|\left\{q_{m}\right\},\;M)=\prod_{m=0}^{M}P(D_{m}|q_{m-1},\;q_{m},\;M)$$

with *q*_− 1_ = −1, *q*_*M*_ = *T* − 1.

#### 2.5.2. Prior on {*q*_*m*_}

Since we have no preferences for any segment boundary configuration other than they be totally ordered, the segment configuration prior becomes

(7) $$P\left(\left\{q_{m}\right\}|M\right)={\left(\begin{array}{c} T-1\\ M \end{array}\right)}^{-1}$$

where $\left(\begin{array}{c} T-1\\ M \end{array}\right)$ is the number of configurations in which *M* ordered segment boundaries can be distributed across *T* − 1 places (segment boundary *M* always occupies position *T* − 1, hence there are only *T* − 1 positions left). While this prior expresses no preferences for boundary positions, it is important for complexity control: as long as *T* » *M* (which is typically the case), this prior will decrease exponentially in *M*, thereby punishing models with larger number of segments.

#### 2.5.3. Prior on *M*

We have no preference for any model complexity (i.e., number of segment boundaries), so we let

(8) $$P(M)=\frac{1}{T}$$

since the number of segment boundaries *M* must be 0 ≤ *M* ≤ *T* − 1.

#### 2.5.4. Posterior of {*q*_*m*_}

For temporal segmentation, the most relevant posterior is that of the {*q*_*m*_} for a given *M*:

(9) $$P(\{q_{m}\}|D,\;M)=\frac{P(D|\{q_{m}\})P(\{q_{m}\}|M)}{P(D|M)}$$

For the denominator, we need to compute *P*(*D*|*M*):

(10) $$P(D|M)=\sum_{q_{0}=0}^{q_{1}-1}\sum_{q_{1}=1}^{q_{2}-1}\ldots \sum_{q_{M-1}=M-1}^{T-1}P(D|\{q_{m}\},\;M)$$

which appears to be $O(T^{M})$ since it involves *M* sums of length O(T). However, using the form of *P*(*D*|{*q*_*m*_}, *M*) (Equation 6) and distributivity of multiplication over addition allows us to “push sums” past all factors which do not depend on the summation variable:

(11) $$\begin{array}{l} P(D|M)=\sum_{q_{0}=0}^{q_{1}-1}\sum_{q_{1}=1}^{q_{2}-1}\ldots \sum_{q_{M-1}=M-1}^{T-1}\prod_{m=0}^{M}P(D_{m}|q_{m-1},\;q_{m})\\ \;=\sum_{q_{0}=0}^{q_{1}-1}P(D_{0}|q_{-1},\;q_{0})\sum_{q_{1}=1}^{q_{2}-1}P(D_{1}|q_{0},\;q_{1})\ldots \\ \;\ldots \sum_{q_{M-1}=M-1}^{T-1}P(D_{M}|q_{m-1},\;q_{M}) \end{array}$$

Each sum of length O(T) needs to be evaluated O(T) times for the possible values of the upper summation boundary. As there are *M* sums, this calculation has complexity $O(MT^{2})$, rather than the naïve $O(T^{M})$. This is an instance of the **sum-product** algorithm (Kschischang et al., 2001). As explained in Endres and Földiák (2005), the expectations of functions of the model parameters (e.g., segment boundary position, segment width or probability of a segment boundary at a given point in time) can be evaluated similarly, if the function depends only on the parameters of one segment.

#### 2.5.5. Observation models *P*(*D*|{*q*_*m*_}) for wrist trajectories

For the wrist trajectories, we employed a multivariate Gaussian observation model with polynomial time-dependence of the mean, because we would like to explore the relationship between power-laws and polynomials. With this choice, we can specify a conjugate prior on the parameters, which allows for an evaluation of expectations and marginal probabilities within each segment in closed form. A prior is conjugate to an observation model, if the resulting posterior has the same functional form as the prior (Bishop, 2007). In that case, posterior updates reduce to parameter updates of the prior, instead of having to compute a (often intractable) multi-dimensional integral. Thus, we can efficiently compute the marginal probability of the data given the number of bin boundaries (Equation 11), as explained above.

The exponential family conjugate prior on the mean μ and the precision matrix **P** (inverse covariance) is given by an extended Gauss–Wishart density (see e.g., Bishop, 2007). Let ${\vec{X}}_{t}\in D$ be a *L* = 3-dimensional vector of wrist positions at time *t* ∈ *T*_*m*_, and *S* be the chosen polynomial order. Let *t*_*m*_ = Δ*t q*_*m* − 1_ be the start time of segment *m*. Then

(12) $$p({\vec{X}}_{t}|t\in T_{m})=N(\vec{X}(t);\;{\vec{\mu }}_{m},\;P_{m}^{-1})$$

(13) $$p(P_{m}|\nu_{m},\;V_{m})=W(P_{m};\;\nu_{m},\;V_{m})$$

(14) $${\vec{\mu }}_{m}=\sum_{i=0}^{S}{\vec{a}}_{i,\;m}{\left(t-t_{m}\right)}^{i}$$

The ${\vec{a}}_{m}=({\vec{a}}_{i,\;m})$ are the polynomial coefficients in segment *m*. Note that this vector has (*S* + 1) · *L* components. $N(\vec{X},\;\vec{\mu },\;\Sigma =P^{-1})$ is a multivariate Gaussian density in $\vec{X}$ with means $\vec{\mu }$ and covariance matrix Σ. W(P; ν, V) is a Wishart density in **P** with ν degrees of freedom and scale matrix **V**. To construct a prior which is conjugate to the likelihood (Equation 12), we choose a vector ${\vec{\alpha }}_{m}=({\vec{\alpha }}_{i,\;m})$ with (*S* + 1) · *L* components, which are the biases on ${\vec{a}}_{m}$. Furthermore, we introduce a symmetric, positive (semi-)definite (*S* + 1) × (*S* + 1) matrix **B**_*m*_, which contains the concentration parameters on ${\vec{a}}_{m}$. The prior on ${\vec{a}}_{m}$ given **P**_*m*_ is then a multivariate Gaussian density

(15) $$p({\vec{a}}_{m}|{\vec{\alpha }}_{m},\;B_{m},\;P_{m})=N({\vec{a}}_{m};\;{\vec{\alpha }}_{m},\;Q_{m}^{-1})$$

where the (*S* + 1)*L* × (*S* + 1)*L* matrix **Q**_*m*_ is given by the Kronecker-product of **B**_*m*_ and **P**_*m*_ (i.e., block-wise multiplication of the entries **B**_*m*, *i*, *j*_ of **B**_*m*_ with **P**_*m*_):

(16) $$Q_{m}=B_{m}⊗P_{m}=\left(\begin{array}{ccc} B_{m,\;0,\;0}P_{m} & \cdots & B_{m,\;0,\;S}P_{m}\\ \vdots & \ddots & \vdots \\ B_{m,\;S,\;0}P_{m} & \cdots & B_{m,\;S,\;S}P_{m} \end{array}\right)$$

It is shown in Endres et al. (2011a) that the product of the Gaussian (Equation 15) with the Wishart (Equation 13) does constitute a conjugate prior on the likelihood given by Equation (12). Since the prior is conjugate, we can evaluate the marginal likelihood of the data in each segment, and BB can be applied with this observation model.

## 3. Results

### 3.1. Trajectory fitting and polynomial order determination

**Figure 3:**
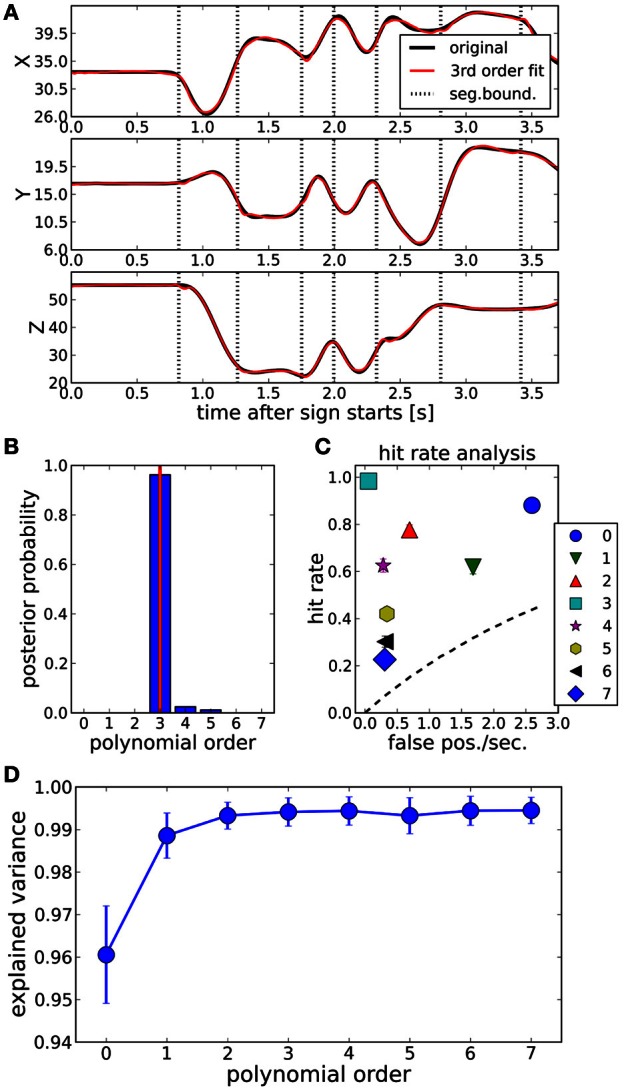
**(A)** Fitting a sign language trajectory (black lines) with a 3rd order polynomial segment model (
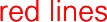
). Dotted vertical lines: most probable segmentation points determined by Bayesian binning. The fit closely models the original trajectory. Explained variance averaged across the whole dataset is >99%, see **(D)**. **(B)** Posterior probability of segment order, computed by using the 3rd order fitted trajectories computed with BB [red lines in panel **(A)**] as data. The correct polynomial (3, indicated by red vertical line) order is recovered with near certainty. **(C)** Hit rate analysis for polynomial segment orders between 0 and 7, using the 3rd order fitted trajectories as data. Dashed line: line of no discrimination. At order 3, hit rate is maximal with no false positives. Error bars (standard errors of the means of hit rate and false positive rate) are smaller than the symbols. **(D)** Explained variance of the original trajectories as a function of the polynomial order of the BB fit. Error bars are ±1 standard deviation, computed across the whole dataset. All polynomial orders are able to fit the data well. For details, see text.

To illustrate that BB is a suitable tool for the computation of compact and accurate ISL trajectory representations, we generated ISL-like trajectories with a 3rd order polynomial segment structure and evaluated if BB was able to recover this polynomial order and the segment boundaries. These trajectories were computed by fitting the original data (black lines in Figure 3A) with 3rd order polynomials using BB and evaluating the posterior expected trajectories (red lines in Figure 3A), which we will refer to as “fitted trajectories.” The dotted vertical lines in this plot are the most probable segmentation points determined by BB, of which it suggests *M* = 7 boundary points with almost certainty. We determined this number by finding the maximum of Equation (11) with respect to *M*.

We then tested whether BB would be able to recover the polynomial order of such fitted trajectories. To this end, we ran BB on the fitted trajectories and evaluated the posterior distribution of the segment order. The result, averaged across the whole dataset, is shown in Figure 3B. The correct polynomial order, here 3, is recovered with near certainty.

The fitted trajectories follow the original trajectories very accurately. Figure 3D shows the variance explained (EV) by polynomial orders between 0 and 7. Even for 0th order fits, EV > 0.95, obtained with on average *M* = 13 bin boundaries. EV > 0.99 for orders greater than 1, and it stays in that range for all tested orders up to 7, where an average *M* = 2 are needed to fit the data.

For a quantitative evaluation of the match between the segmentation points of the fitted trajectories, and the BB segmentation points computed on these fitted trajectories, we conducted a hit rate analysis similar to Endres et al. (2011a). The results are plotted in Figure 3C. We obtained this plot in the following way: after computing the most probable number of segmentation point with Equation (11), say *M*_opt_, we found the *M*_opt_ maxima of the posterior distribution of the segmentation point locations. This yielded the “predicted segmentation points” (PSP). Denote with PSP_3_ the segmentation points of the fitted trajectory, and with PSP′_*k*_ the segmentation points of a *k*-th order BB model computed from the fitted trajectory. A PSP′_*k*_ counted as a hit if it was within an accuracy window of 90 ms of a PSP_3_, and if no other PSP′_*k*_ had been matched to that PSP_3_ already. All remaining PSP′_*k*_ comprised the false positives. PSP_3_s without a matching PSP′_*k*_ were counted as misses. The hit rate is then computed in the usual way:

$$\text{hit rate}=\frac{\text{hits}}{\text{hits}+\text{misses}}$$

which implies that the hit rate ≤1. Moreover, the miss rate (or false negative rate) is just given by miss rate = 1 − hit rate. Computing a false positive rate for a standard ROC analysis

$$\text{false positive rate}=\frac{\text{false positives}}{\text{false positives}+\text{true negatives}}$$

is somewhat problematic, since it requires the evaluation of the “true negatives, ” i.e., the number of instances where neither BB model predicts a segmentation event. This number depends on the chosen discretization: the false positive rate can be reduced almost arbitrarily by increasing the temporal resolution, since both PSP′_*k*_ and PSP_3_ are (almost) point events. We therefore chose to evaluate the false positives per second, which is largely independent of the temporal resolution. As a reference, we computed a “line of no discrimination” (dashed line in Figure 3C) assuming a homogeneous Poisson process with rate parameter λ as a generator of uninformative segmentation events. Each setting of λ corresponds to one point on the line of no discrimination. If a given model's performance is above that line, then it can be said to provide an informative signal about the fitted trajectory segmentation points.

All polynomial orders provide an informative signal, with the 3rd order model performing optimally: it combines a very high hit rate with almost no false positives.

We performed the above analyses with fitted trajectories of orders between 1 and 7, see Appendix A1. The results are very similar to the 3rd order results presented here: the posterior distribution of the segment order peaks strongly at the order of the fitted trajectories. Moreover, the hit rates of the fitted order are near one, with almost no false positives.

### 3.2. Power law ground truth evaluation

We applied BB to the generated power law ground truth trajectories to determine whether the segments predicted by BB would match the imposed power law segments [see section 2.4]. Prior hyperparameters were ${\vec{\alpha }}_{m}=\vec{0}$, **B**_*m*_ = δ(*i*, *j*) × 0.1, prior covariance was diagonal with the data variances as diagonal entries. We chose *T* = 100 time discretization points, and experimented with polynomial orders between 0 (constant trajectories per segment) and 7.

**Figure 4:**
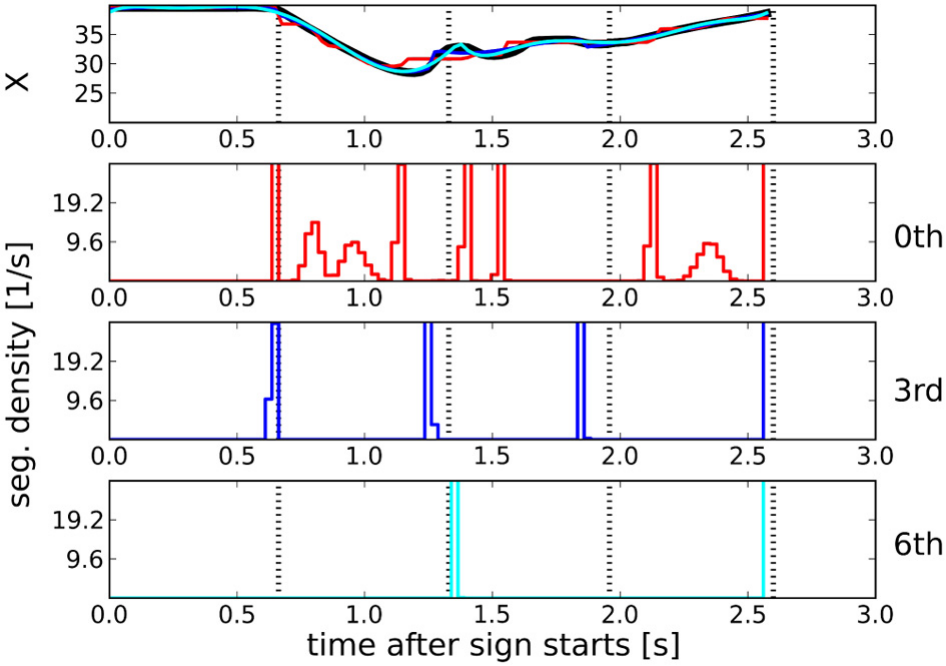
**Top panel:** Trajectory of X coordinate (black line) and posterior expected trajectories for observation models of 0th (red), 3rd (blue), and 6th (cyan) order. All observation models provide a good fit. Vertical dotted black lines indicate ground truth segmentation points in all panels. **Lower panels**: Segmentation densities (i.e., probability density of segmentation boundaries) for these observation models. The 3rd order model puts boundaries close to the ground truth, with no false positives in this example.

As shown in Figure 4, top panel, the posterior expectation of the trajectories follows the actual trajectory closely for all orders. However, the 0th order observation model requires a large number of segments to do so. With increasing order, the number of necessary segments decreases. Shown in Figure 4, lower panels, are the segmentation densities, i.e., the posterior probability densities of finding a segment boundary at a given point in time. Black dotted vertical lines indicate ground truth segmentation boundaries in all panels. The 0th order model generates many, uncertain segmentation boundaries, resulting in a large number of false positives with respect to the ground truth. The 6th order model generates too few segments, but its segment boundaries coincide with the ground truth. The 3rd order model puts boundaries close to the ground truth, without false positives in this example.

**Figure 5:**
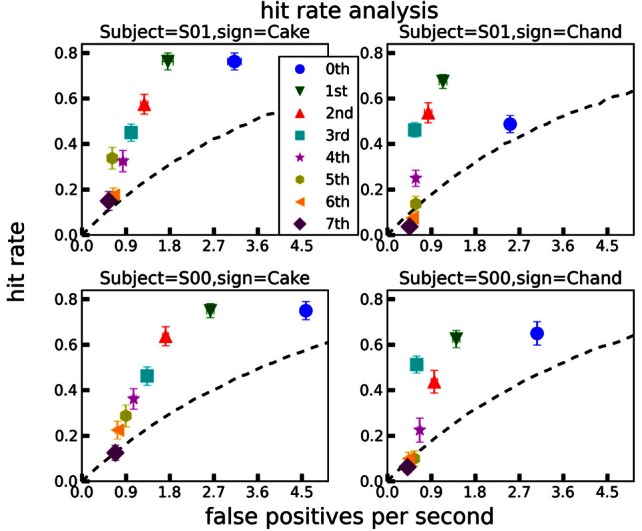
**Hit rate analyses for each sign (“cake” and “chandelier”) and subject (“S00” and “S01”), for all polynomial orders between 0 and 7.** A predicted segmentation point counted as a “hit” if it occurred within an accuracy window of 90 ms around a ground truth segmentation point. The lines of no discrimination (black dashed) were computed assuming a homogeneous Poisson process as a generator of uninformative segmentation events. Error bars are ±1 standard errors of the means. For details, see text.

**Figure 6:**
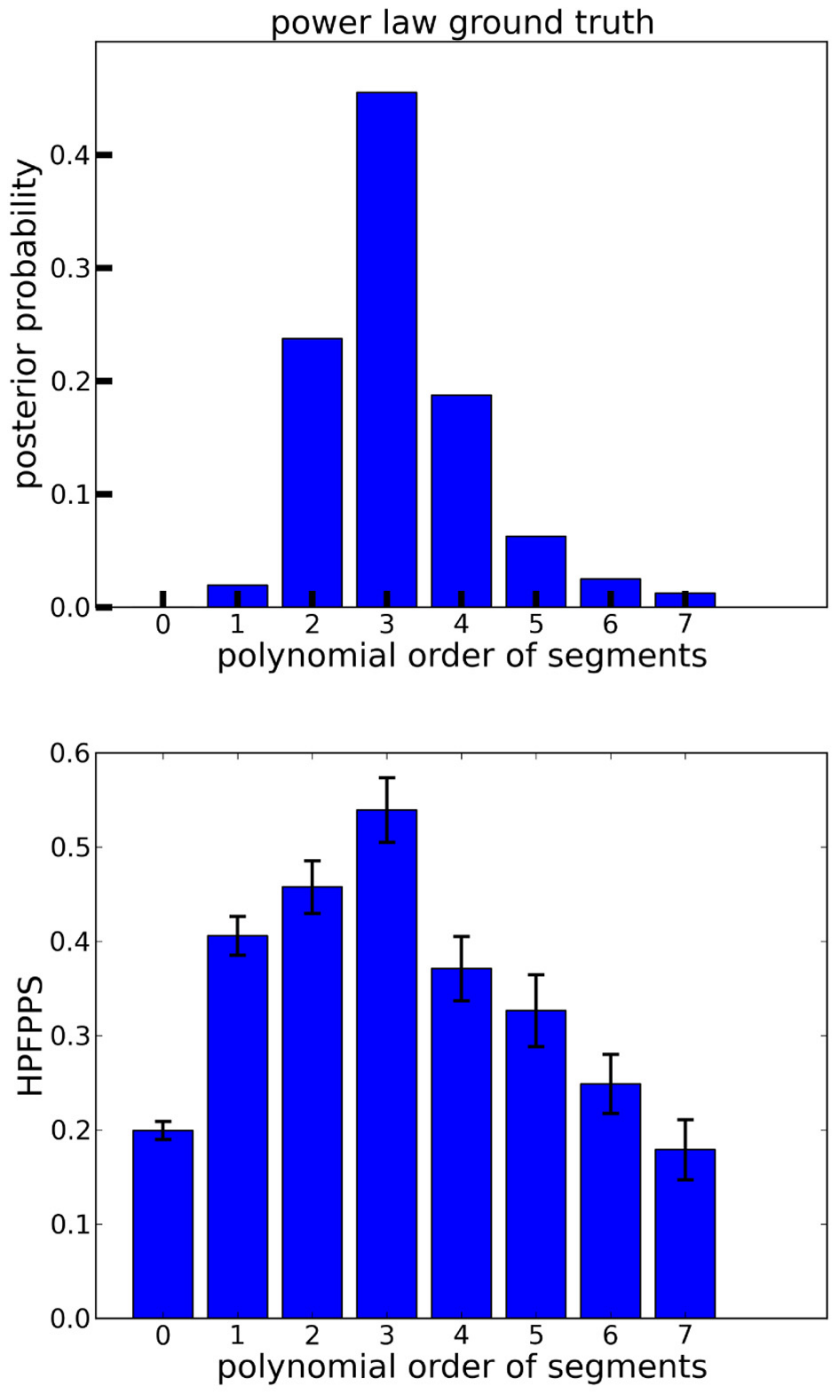
**Polynomial order evaluation of power law ground truth data. Top panel**: Marginal posterior probabilities of observation models with polynomial orders between 0 and 7. Posteriors were averaged across signs and subjects. Order 3 is most probable. **Bottom panel**: Hit rate per false positives per second (HPFPPS) as a function of the polynomial order of the segments. The higher HPFPPS, the fewer false positives are incurred for each hit. Hence, a high HPFPPS is preferable. In the power law ground truth data of Figure 5, this quantity is maximized for order 3. Error bars are ±1 standard errors of the means.

For a more quantitative performance evaluation, we conducted a hit rate analysis as described above. The results are plotted in Figure 5. Here, power law ground truth segmentation points (vertical dotted lines in Figure 4) are compared against segmentation points predicted by BB models of polynomial orders between 0 and 7. The BB segmentation points were obtained as described in section 3.1. As can be seen in Figure 5, most polynomial orders provide an informative signal about the ground truth. However, the lower orders generate significantly more false positives per second than the higher ones. For orders >3, the hit rate decreases without a matching decrease in the false positives. This can be seen more clearly in the hit rate per false positives per second (HPFPPS) plot in Figure 6, bottom panel. Let

(17) $$\text{HPFPPS}=\frac{\text{hit rate}}{\text{false positives per second}}$$

The larger HPFPPS, the fewer false positives are incurred per hit, hence a large HPFPPS is desirable. In the ground truth data, it peaks at polynomial segment order 3. This peaking is significant (Kruskal–Wallis, *p*<10^−7^ for both testing order 3 vs. rest and testing all orders against each other). A polynomial order of ≈3 therefore seems a reasonable choice for these data. This observation is confirmed by the posterior distribution of the polynomial orders (Figure 6, top panel), which peaks at order 3 for the ground truth data.

### 3.3. Polynomial order of ISL trajectories

**Figure 7:**
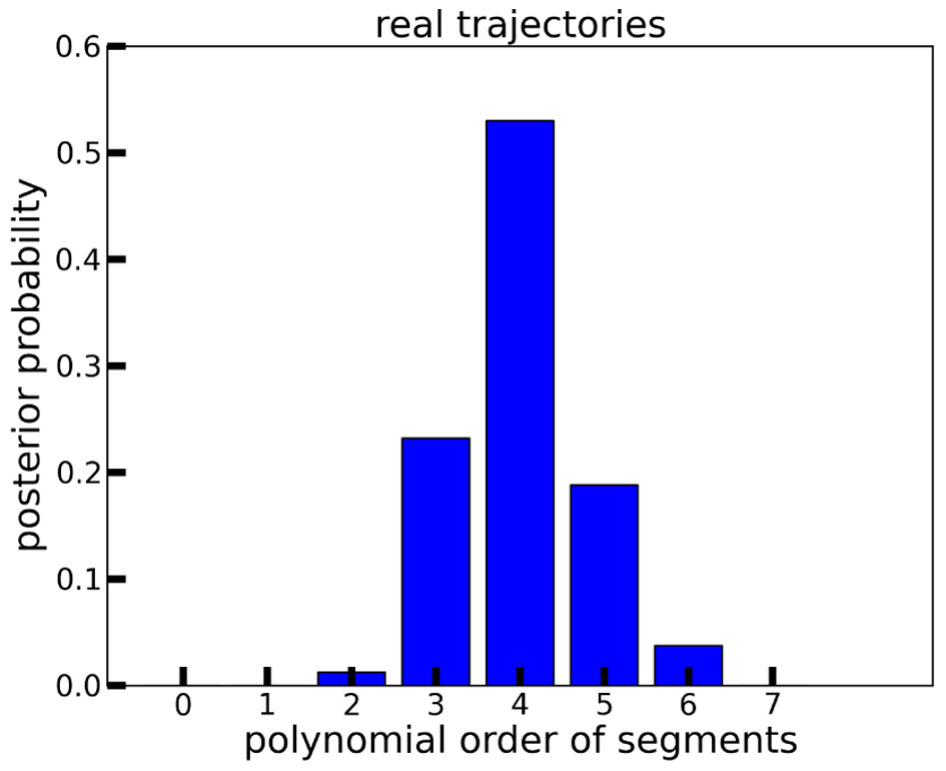
**Marginal posterior probabilities of observation models with polynomial orders between 0 and 7, computed on the real ISL trajectories.** Posteriors were averaged across signs and subjects. 4th order is preferred.

Interestingly, the best polynomial order for the real ISL data peaks at 4, with *P*(3 ≤ order ≤ 5) > 0.95 (see Figure 7). The order of 5 corresponds to trajectories that comply with the minimum jerk principle (Flash and Hogan, 1985), which has been largely established as describing the structure of many types of natural movements (Todorov, 2004). This shows that the power law temporal structure in the real data requires higher order polynomials, suggesting that the co-articulation between consecutive power law segments is better represented by polynomial orders > 3. In other words, BB combined with a segment-wise polynomial trajectory model results in biologically reasonable segments that could be indicative of individual optimally controlled submovements. We elaborate this point further in the discussion (section 4).

### 3.4. Interpretation of segments

We worked on single signs, so the segments discovered by BB are units on a sub-semantic level. Even within movements, like a drawing of a letter or an ellipse, there are often multiple segments that are described by different mixtures of several non-euclidian geometries (Bennequin et al., 2009; Polyakov et al., 2009a; Pham and Bennequin, 2012). Our approach aimed at estimating such invariants. Consequently, a hit rate analysis for the real data cannot be done meaningfully, because we segmented single signs and because there is no accepted method for the sequential decomposition of trajectories based on power laws. To find out the relevant segments is exactly the scientific problem in motor control research which is addressed by our Bayesian approach. We therefore created ground truth data with known segments against which the Bayesian decomposition was successfully compared. Whether these segments are related to the temporal aspects of “phonemes” of sign language (Sandler and Lillo-Martin, 2006) (phonemes are defined as the smallest, contrastive units in a spoken language) remains to be investigated.

## 4. Discussion

We presented two novel contributions in this paper: firstly, we demonstrated the applicability of BB with piecewise polynomial observation models to motion capture data with a segment-wise power law structure. Secondly, we found that ISL wrist trajectories are best described by observation models with polynomial orders between 3 and 5.

This is compatible with established principles in motor control, like the minimum jerk and minimum acceleration principles. The study in Richardson and Flash (2002) suggested three main insights. First, among all optimization criteria whose mean squared derivative (MSD) cost functions are

$$C_{n}=\int_{0}^{T}{\left|\left|\frac{d^{n}r}{dt^{n}}\right|\right|}^{2}dt,$$

the optimal trajectories that correspond to *n* = 3 (minimum jerk) provide the best kinematic fit to point-to-point reaching movements. Second, for periodic movements, the cost functions corresponding to *n* = 3 (minimum jerk—fifth order polynomials) and *n* = 4 (minimum snap—seventh order polynomials) provide reasonable predictions while optimal trajectories corresponding to the limit case *n* → ∞ converge to the 2/3 power law. Third, earlier studies (e.g., Viviani and Cenzato, 1985) have suggested based on the two-thirds power law that complex movements should be segmented at inflection points, however, this segmentation criterion is also predicted by a path-constrained minimum jerk criterion and thus may not necessarily be a result of segmented control by the brain. It should be noted that inflection points are special cases in equi-affine geometry since at these points the equi-affine arclength vanishes faster than the Euclidean arclength [$\frac{d\sigma }{ds}\to 0$, see Flash and Handzel (2007); Bennequin et al. (2009)] from which one deduces that that the 2/3 power law breaks down at inflection points. Hence any kinematic model that is compatible with the 2/3 power law will give similar segments to those hypothesized according to the law and this explains the observations in Richardson and Flash (2002) and of Todorov and Jordan (Todorov and Jordan, 1998)—whereby both studies were using a constrained minimum jerk (as it was named by Todorov and Jordan). However, it was not *a-priori* clear whether this agreement will hold for different complex geometries and for different optimization principles. Our results indicate that the two approaches lead to compatible segmentations in a general sense. The unsupervised BB approach shows that for highly complex motor tasks, optimal MSD segments are temporally aligned with the generalized power law segments. It should be noted that MSD criteria are drawn from first principles and thus provide a predictive model while the two thirds power law was mainly studied as a descriptive model (Lacquaniti et al., 1983; Viviani and Cenzato, 1985). Nevertheless, it was found that the 2/3 power law is theoretically founded in equi-affine geometry (Pollick and Sapiro, 1996; Flash and Handzel, 2007; Bennequin et al., 2009). From this we hypothesize that the power law modulation is a possible outcome of an optimization procedure that takes into account different MSD criteria such as minimum acceleration, minimum jerk and minimum snap models.

Another implication is related to the distribution of polynomial orders found in the power law ground truth kinematics vs. that of the original kinematics. The kinematics in the ground truth dataset was implemented by introducing a perfect power law segmentation that respects the timing in the original data in a segment-wise manner and maintains continuity at the boundaries of segments. The original kinematics may differ from the ground truth in the parameters of the natural power law regularities and the transitions between segments which may be comprised of both co-articulatory movement and movement kinematics not adhering to the power law. The latter is more probably capturing the differences between the two datasets. We therefore hypothesize that the differences in the distribution of the polynomial orders found by BB for these two datasets is related to transitional movements that are less compatible with the generalized power law and require a description involving higher order polynomials. The interpretation of this result in terms of the MSD approach suggests that the minimum acceleration model (*C*_2_) does not provide an equally good explanation for the complex co-articulatory movements in between and at the boundaries of power law segments for which jerk and snap minimizations are required.

Three methods for motion capture data segmentation are compared in Barbič et al. (2004): segment-wise PCA, probabilistic PCA (pPCA) and finite Gaussian mixture models. The pPCA methods is found to deliver the best performance compared to manual segmentation. Our 0th order polynomial segment model, due to its full covariance matrix, essentially describes each segment by a different (p)PCA decomposition. This decomposition could be extracted from the posterior covariance matrices. Hence, the 0th order model is approximately equivalent to the best method of Barbič et al. (2004). Segment positions are decided via a Mahalanobis distance criterion in Barbič et al. (2004), which is related, but not equivalent to the marginal log-likelihood of our Gaussian observation model used by BB^2^. As illustrated in Figure 5, our higher-order models offer a significant performance advantage over a pPCA model with constant means on the ISL data.

The authors of Polyakov et al. (2009a) found that monkey scribbling trajectories could be fitted well with parabolic pieces. Such pieces can be generated by our 2nd order segment model. We showed that higher polynomial orders are favored on both an ISL-inspired ground truth and real (human) ISL data. However, the segmentation criteria in that paper appears rather different from ours: while we use a marginal likelihood based criterion which follows from the polynomial observation model, the authors of Polyakov et al. (2009b) first extracted from the recorded data portions corresponding to active movement and others of rest. The extracted movement portions were segmented into strokes at curvature extrema. This is just one example of a wide range of segmentation approaches based on kinematic descriptors, another example is the work of Fod (2002) which uses speed features.

Hidden Markov Models (HMM) have been used extensively for both action segmentation and recognition, see e.g., Kulic et al. (2009) for a template based approach, or the switching HMM approach of Green (2003) where actions are segmented into “dynemes,” a kind of dynamical primitives. While dynamical primitives are in principle more invariant, and hence variation tolerant (e.g., against time-warping) than polynomial segments, they are also much harder to learn: in Green (2003), the dynemes had to be defined manually. For American Sign Language recognition, Vogler and Metaxas (1998) used a semi-supervised training scheme, which was extended to deal with two-hand signing using parallel HMMs in Vogler and Metaxas (1999, 2001). In that work, labeled and pre-segmented data were used to bootstrap the training process. In contrast, we segmented sign language based on kinematic regularities which, in order to be independent of representation or a linguistic formalism (Sandler and Lillo-Martin, 2006), must be unsupervised. Furthermore, unsupervised segmentation facilitates working with large datasets.

We conclude that BB combined with polynomial observation models represents a biologically well-inspired way for the unsupervised extraction of movement primitives from natural action streams. It remains to be investigated whether our approach is applicable to data obtained with other recording modalities, e.g., EMG, and if it yields interpretable results on forces/torques instead of positions. Instead of a polynomial observation model for (wrist) positions, one could also construct an observation model for velocities and curvatures. This would lead to a more direct power law segmentation than the approach presented here, and will be of interest for future work.

### Conflict of interest statement

The authors declare that the research was conducted in the absence of any commercial or financial relationships that could be construed as a potential conflict of interest.

## Figures and Tables

**Figure A1:**
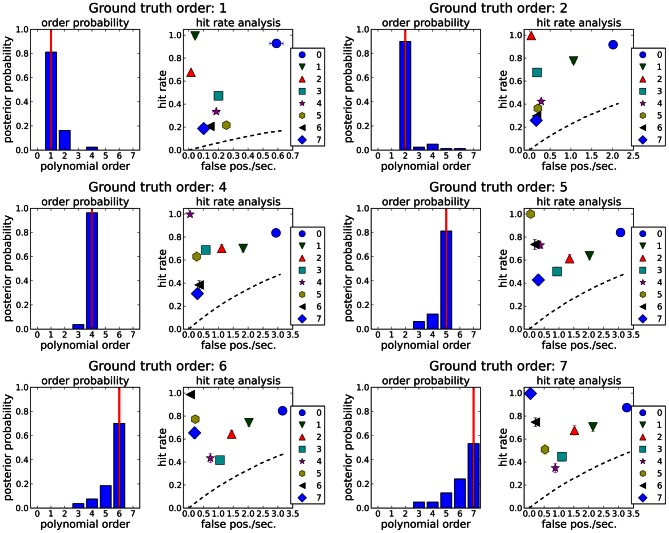
**Order posteriors and hit rate analyses for polynomial segment orders between 0 and 7, using fitted trajectories of several orders as ground truth.** Vertical red lines in the order posteriors indicate ground truth order. Dashed lines in the hit rate analyses: line of no discrimination. Results for ground truth order 3 were depicted above in Figure 3C. Order posteriors are maximal at the respective ground truth orders, i.e., the correct order is recovered by BB with high probability. Hit rates are near one, with almost no false positives if and only if the ground truth order and BB order match. Error bars (standard errors of the means of hit rate and false positive rate) are mostly smaller than the symbols.

## Footnotes

^1^E.g., choose Δ*t* in the order of 1/sampling rate.

^2^Due to the conjugate prior on the polynomial observation model, we can integrate over the likelihood before taking the logarithm, see Endres et al. (2011a) for details. The resulting marginal log-likelihood is monotonically related to a Mahalanobis distance.

## A. Appendix

### A.1. Polynomial order determination for ground truth orders ≠ 3

In response to a reviewer request, we repeated the analysis in section 3.1 for polynomial ground truth orders other than 3. The resulting order posteriors and hit rate analyses are depicted in Figure A1. For all tested ground truth orders, the BB order posterior peaks at the ground truth order, i.e., this order can be recovered with high probability. Furthermore, hit rate is near one with almost no false positives for the ground truth order only, i.e., correct segment boundaries can also be determined by BB.
